# Supplementary figures and images for: Malaria incidence rose following the introduction of neonicotinoid-based IRS in selected districts in northern Ghana: An observational analysis
Source: PLOS Glob Public Health. 2026 Apr 17;6(4):e0005267. doi: 10.1371/journal.pgph.0005267 (PMC13089701; doi:10.1371/journal.pgph.0005267)

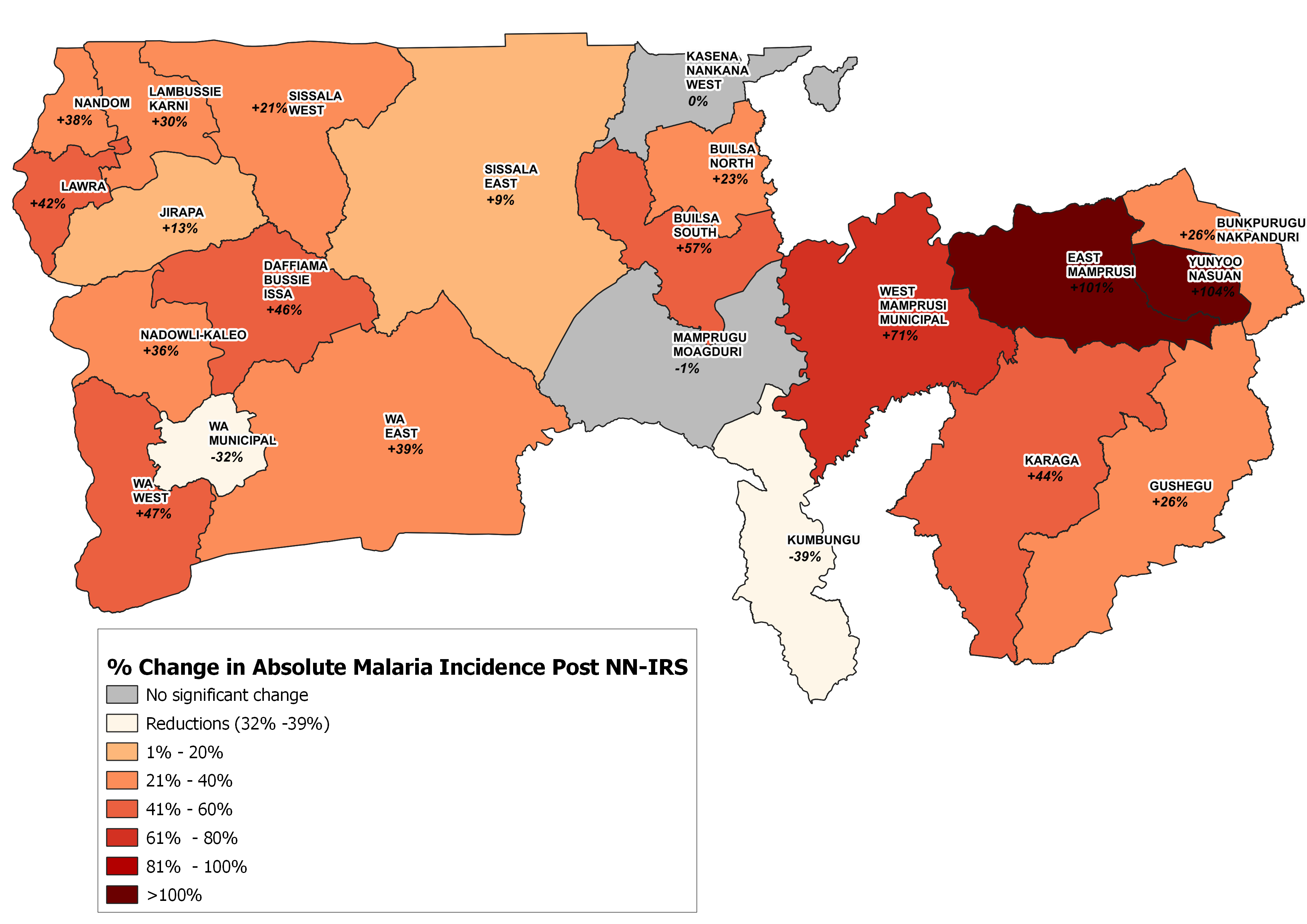

Supplement: S1 Fig — (TIFF) [file pgph.0005267.s001.tiff]

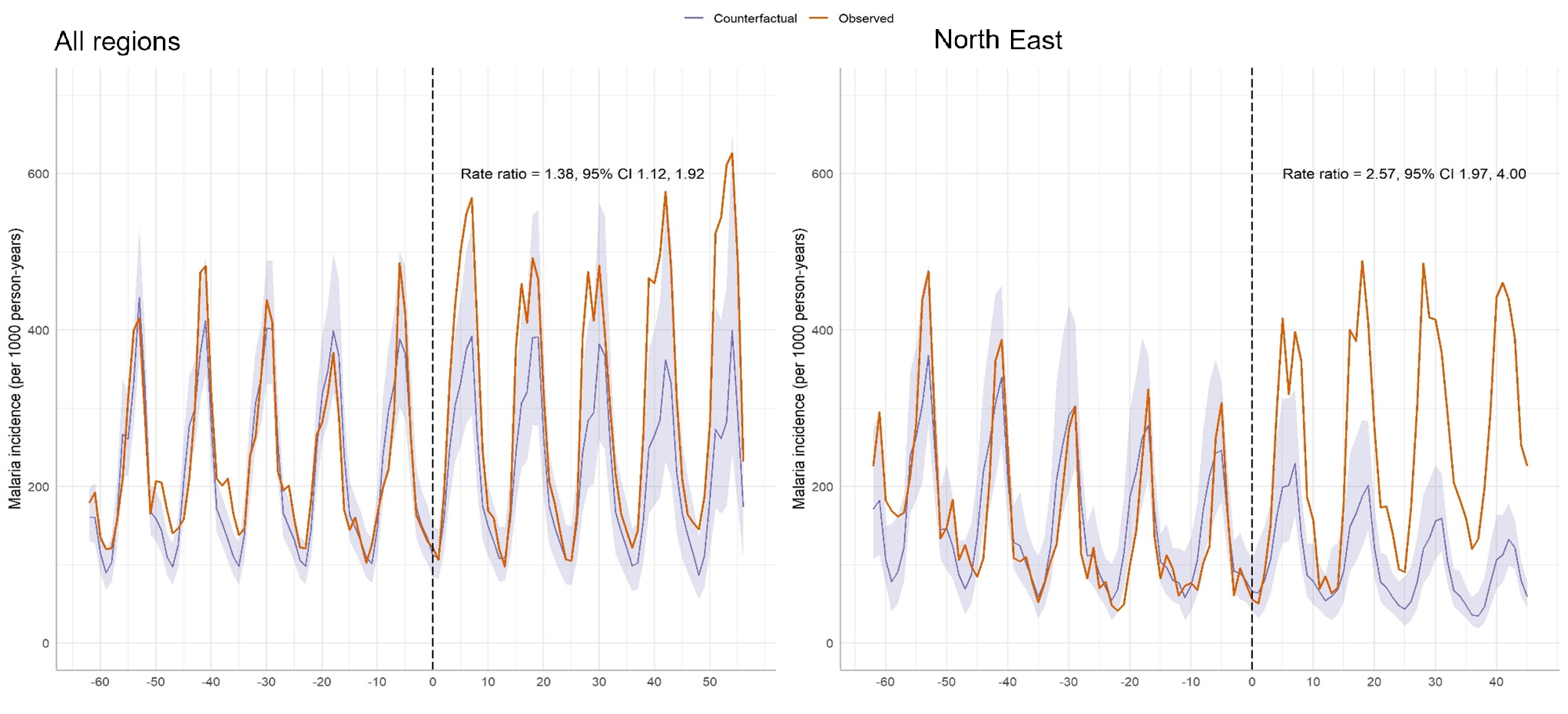

Supplement: S2 Fig — (TIF) [file pgph.0005267.s002.tif]

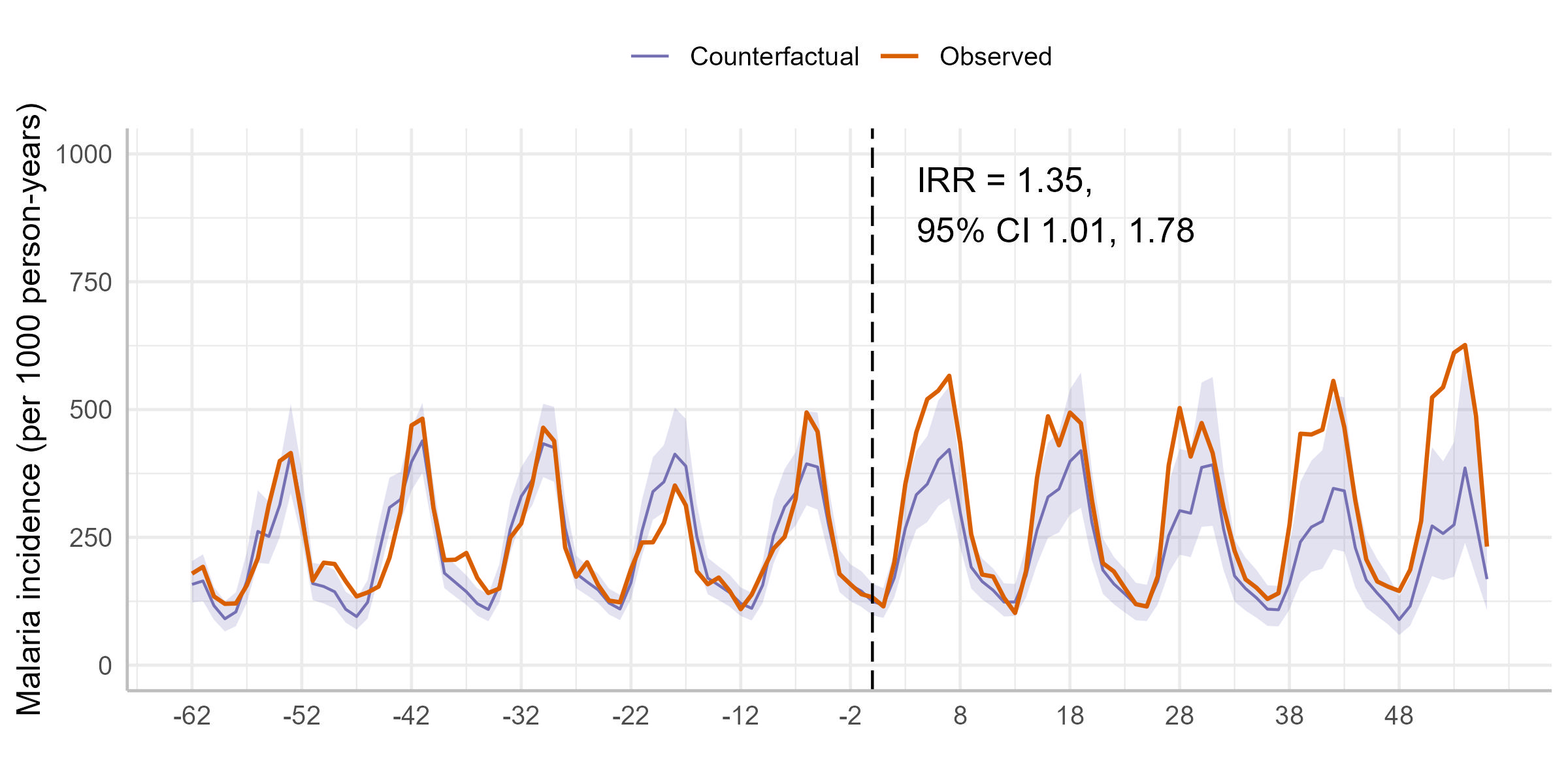

Supplement: S3 Fig — (JPG) [file pgph.0005267.s003.jpg]

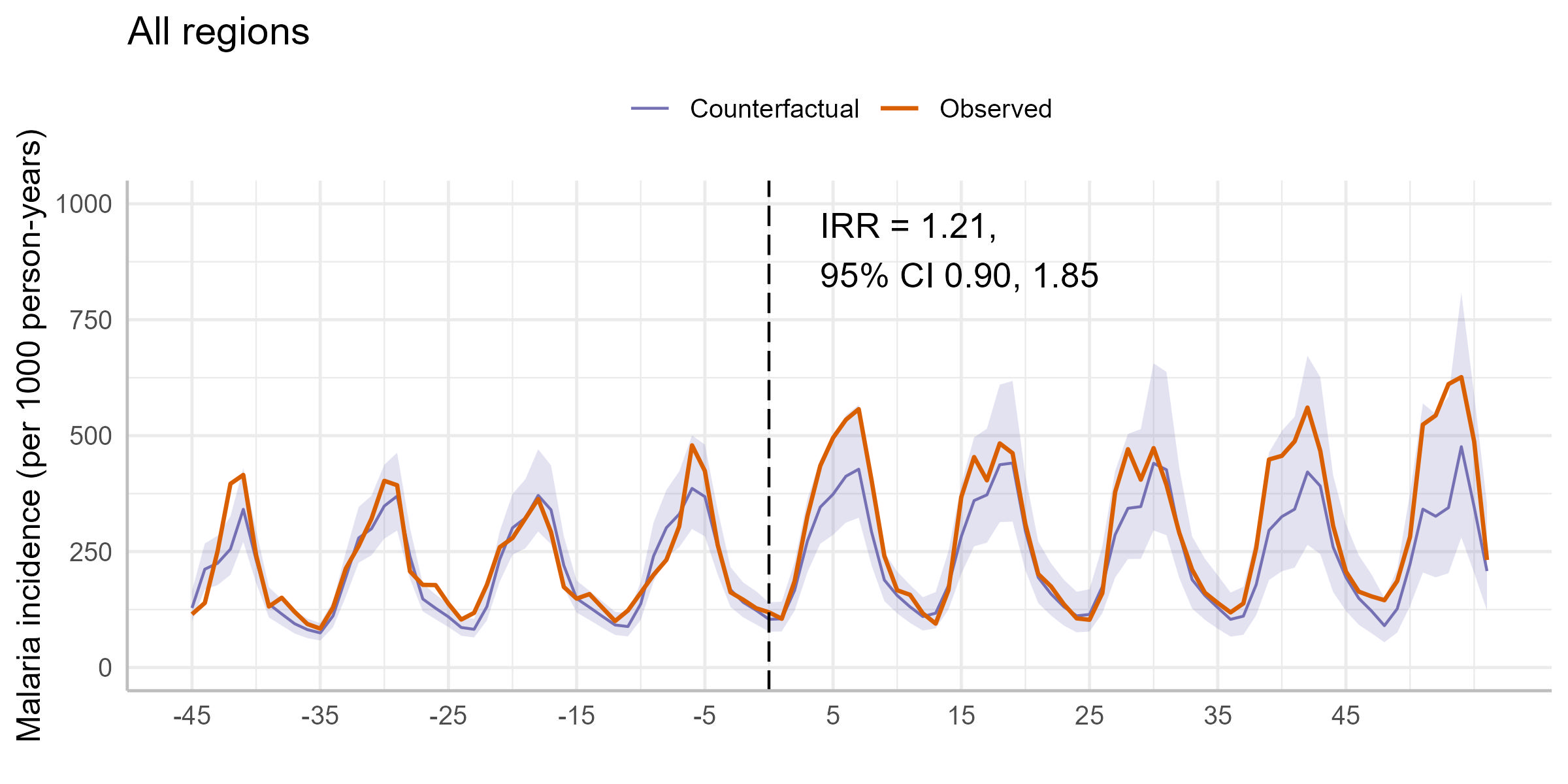

Supplement: S4 Fig — (JPG) [file pgph.0005267.s004.jpg]

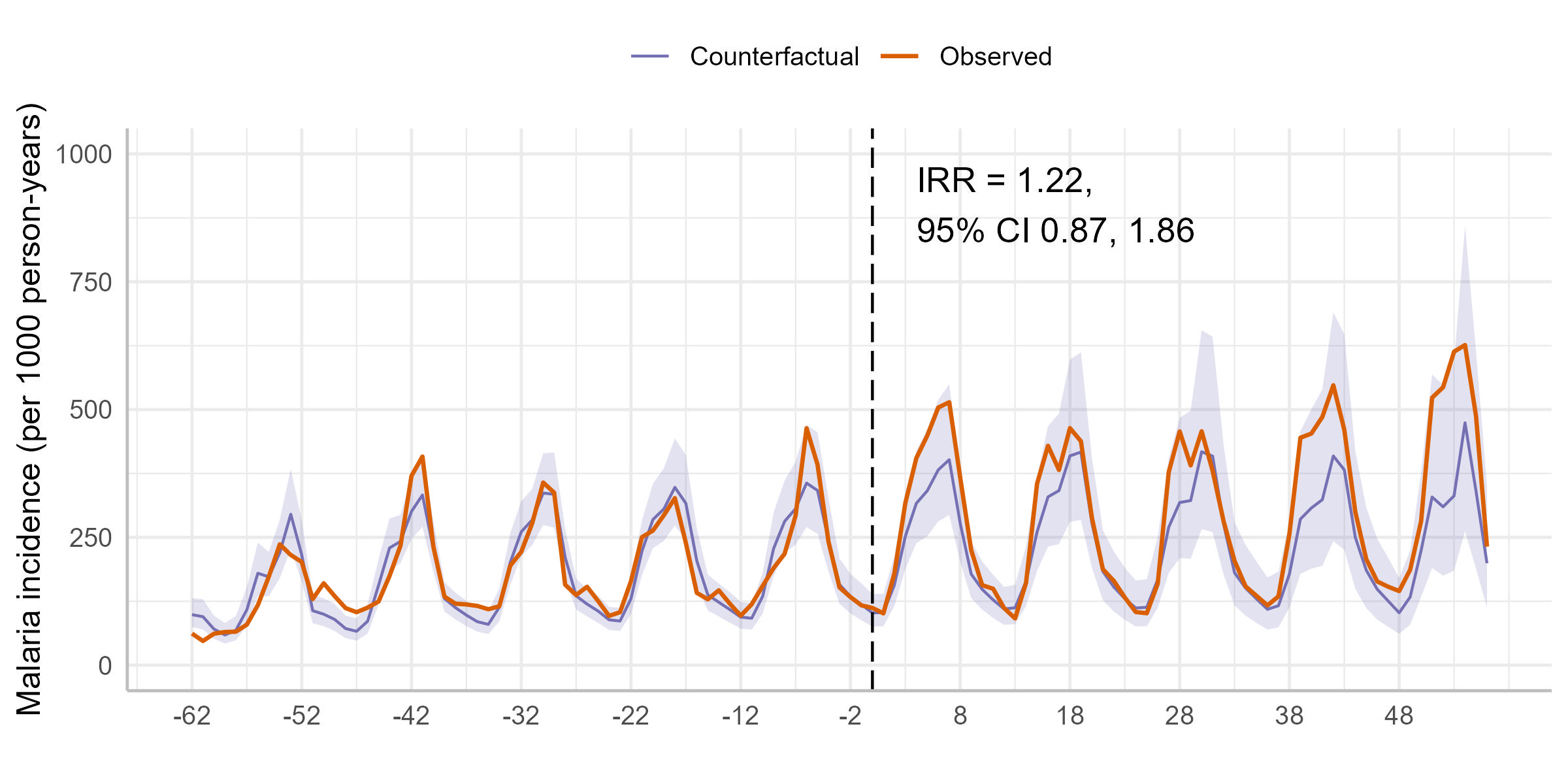

Supplement: S5 Fig — (JPG) [file pgph.0005267.s005.jpg]
