# Supplementary material for: Malaria incidence rose following the introduction of neonicotinoid-based IRS in selected districts in northern Ghana: An observational analysis
Source: PLOS Glob Public Health. 2026 Apr 17;6(4):e0005267. doi: 10.1371/journal.pgph.0005267 (PMC13089701; doi:10.1371/journal.pgph.0005267)
Supplement: S1 Table — (DOCX) [file pgph.0005267.s006.docx]

**S1 Table. Summary of type of insecticides used in our study districts from 2008-2022**

| **Region** | **Districts** | **IRS start year** | **2008** | **2009** | **2010** | **2011** | **2012** | **2013** | **2014** | **2015** | **2016*^‡^*** | **2017** | **2018** | **2019** | **2020** | **2021** | **2022** |
| --- | --- | --- | --- | --- | --- | --- | --- | --- | --- | --- | --- | --- | --- | --- | --- | --- | --- |
| North  East | Bunkpurugu -Nakpanduri | 2011 | Nsp | Nsp | Nsp | PY | PY | PY | OP | OP | OP | OP | OP | OP | NN | NN | NN |
|  | East Mamprusi | 2009 | Nsp | PY | PY | PY | PY | PY | OP | OP | OP | OP | OP | NN | NN | NN | NN |
|  | Mamprugu-Moagduri | 2008 | PY | PY | PY | PY | PY | PY | OP | OP | OP | OP | NN | NN | OP | NN | NN |
|  | West Mamprusi | 2008 | PY | PY | PY | PY | PY | PY | OP | OP | OP | OP | OP | NN | NN | NN | NN |
|  | Yunyoo-Nasuan | 2011 | Nsp | Nsp | Nsp | PY | PY | PY | OP | OP | OP | OP | OP | OP | NN | NN | NN |
| Northern | Gushiegu***^†^*** | 2008 | PY | PY | PY | PY | PY | Nsp | Nsp | Nsp | Nsp | OP | OP | OP | NN | NN | NN |
|  | Karaga***^†^*** | 2008 | PY | PY | PY | PY | PY | Nsp | Nsp | Nsp | Nsp | OP | OP | OP | NN | NN | NN |
|  | Kumbungu***^†^*** | 2008 | PY | PY | PY | PY | PY | Nsp | Nsp | OP | OP | OP | OP | OP | NN | NN | NN |
| Upper East | Builsa North***** | 2013 | Nsp | Nsp | Nsp | Nsp | Nsp | CAB | OP | Nsp | Nsp | OP | OP | NN | NN | NN | NN |
|  | Builsa South***** | 2013 | Nsp | Nsp | Nsp | Nsp | Nsp | CAB | OP | Nsp | Nsp | OP | OP | NN | NN | NN | NN |
|  | Kasena-Nankana West***** | 2013 | Nsp | Nsp | Nsp | Nsp | Nsp | OP | OP | Nsp | Nsp | OP | OP | NN | NN | NN | NN |
| Upper West | Daffiama-Bussie-Issa | 2012 | Nsp | Nsp | Nsp | Nsp | OP | CAB | Nsp | OP | OP | OP | NN | NN | NN | NN | NN |
|  | Jirapa | 2012 | Nsp | Nsp | Nsp | Nsp | OP | CAB | Nsp | OP | OP | OP | NN | NN | NN | NN | NN |
|  | Lambussie | 2012 | Nsp | Nsp | Nsp | Nsp | OP | CAB | Nsp | OP | OP | OP | OP | NN | NN | NN | NN |
|  | Lawra | 2012 | Nsp | Nsp | Nsp | Nsp | OP | CAB | Nsp | OP | OP | OP | OP | NN | NN | NN | NN |
|  | Nadowli-Kaleo | 2012 | Nsp | Nsp | Nsp | Nsp | OP | CAB | Nsp | OP | OP | OP | NN | NN | NN | NN | NN |
|  | Nandom | 2012 | Nsp | Nsp | Nsp | Nsp | OP | CAB | Nsp | OP | OP | OP | OP | NN | NN | NN | NN |
|  | Sissala East | 2013 | Nsp | Nsp | Nsp | Nsp | Nsp | OP | OP | OP | OP | OP | OP | NN | NN | NN | NN |
|  | Sissala West | 2012 | Nsp | Nsp | Nsp | Nsp | OP | CAB | OP | OP | OP | OP | NN | NN | NN | NN | NN |
|  | Wa East | 2012 | Nsp | Nsp | Nsp | Nsp | OP | CAB | OP | OP | OP | OP | OP | NN | NN | NN | NN |
|  | Wa Municipal | 2012 | Nsp | Nsp | Nsp | Nsp | OP | CAB | Nsp | OP | OP | OP | OP | NN | NN | NN | NN |
|  | Wa West | 2012 | Nsp | Nsp | Nsp | Nsp | OP | CAB | Nsp | OP | OP | OP | OP | NN | NN | NN | NN |
| *Green: PY- Pyrethroids Alpha cypermethrin (and deltamethrin only in 2010). Orange: CAB – Carbamate, mainly propoxur.* *Blue: OP- organophosphates, most commonly Actellic ® 300CS, yellow NN- neonicotinoids, mainly SumiShield® 50WG and Fludora®Fusion WP SB. Nsp=not sprayed.*  ***^†^****IRS was withdrawn in 2012 and* *re-introduced in 2015 in Kumbungu and 2017 in Gushegu and Karaga.*  ***** *IRS was withdrawn from Upper East region in 2015 and re-introduced in Builsa North, Builsa South, and Kasena-Nankana West districts in 2017*  ***^‡^*** *Point mass ITN distribution of pyrethroid only ITNs across all 4 regions in 2016* | | | | | | | | | | | | | | | | | |
