# Supplementary material for: Malaria incidence rose following the introduction of neonicotinoid-based IRS in selected districts in northern Ghana: An observational analysis
Source: PLOS Glob Public Health. 2026 Apr 17;6(4):e0005267. doi: 10.1371/journal.pgph.0005267 (PMC13089701; doi:10.1371/journal.pgph.0005267)
Supplement: S2 Table — (DOCX) [file pgph.0005267.s007.docx]

**S2 Table: Timeline for the introduction of different interventions during the study period**

| **Region** | **Intervention** | **Year of Study and Intervention Mix** | | | | | | | |
| --- | --- | --- | --- | --- | --- | --- | --- | --- | --- |
|  |  | **2015** | **2016** | **2017** | **2018** | **2019** | **2020** | **2021** | **2022** |
| North East | Mass ITN (PY-only) |  | ✔️ |  |  |  |  |  |  |
|  | IRS (OP) | ✔️ | ✔️ | ✔️ | ✔️ | ✔️ |  |  |  |
|  | IRS (NN) |  |  |  |  | ✔️ | ✔️ | ✔️ | ✔️ |
|  | SMC |  |  |  |  | ✔️ | ✔️ | ✔️ | ✔️ |
| Northern***^†^*** | Mass ITN (PY-only) |  | ✔️ |  |  |  |  |  |  |
|  | IRS (OP) | ✔️ | ✔️ | ✔️ | ✔️ | ✔️ |  |  |  |
|  | IRS (NN) |  |  |  |  |  | ✔️ | ✔️ | ✔️ |
|  | SMC |  |  |  |  | ✔️ | ✔️ | ✔️ | ✔️ |
| Upper East* | Mass ITN (PY-only) |  | ✔️ |  |  |  |  |  |  |
|  | IRS (OP) | *Nsp* | *Nsp* | ✔️ | ✔️ | ✔️ |  |  |  |
|  | IRS (NN) |  |  |  |  |  | ✔️ | ✔️ | ✔️ |
|  | SMC |  | ✔️ | ✔️ | ✔️ | ✔️ | ✔️ | ✔️ | ✔️ |
| Upper West | Mass ITN (PY-only) |  | ✔️ |  |  |  |  |  |  |
|  | IRS (OP) | ✔️ | ✔️ | ✔️ | ✔️ | ✔️ |  |  |  |
|  | IRS (NN) |  |  |  |  | ✔️ | ✔️ | ✔️ | ✔️ |
|  | SMC | ✔️ | ✔️ | ✔️ | ✔️ | ✔️ | ✔️ | ✔️ | ✔️ |
| *Grey: Mass ITN distribution; Blue: OP- organophosphates, most commonly Actellic ® 300CS; Yellow: NN- neonicotinoids, mainly SumiShield® 50WG and Fludora®Fusion WP SB. Nsp=not sprayed; Orange: Seasonal malaria chemoprevention (SMC).*  ***^†^*** *IRS was withdrawn in 2012 and* *re-introduced in 2015 in Kumbungu and 2017 in Gushegu and Karaga.*  **IRS was withdrawn from Upper East region in 2015 and* *re-introduced in 2017.* | | | | | | | | | |
