# Supplementary material for: Malaria incidence rose following the introduction of neonicotinoid-based IRS in selected districts in northern Ghana: An observational analysis
Source: PLOS Glob Public Health. 2026 Apr 17;6(4):e0005267. doi: 10.1371/journal.pgph.0005267 (PMC13089701; doi:10.1371/journal.pgph.0005267)
Supplement: S3 Table — (DOCX) [file pgph.0005267.s008.docx]

**S3 Table**. **Pre-and Post-Neonicotinoid IRS Malaria Incidence grouped by districts and region**

| **Region** | **District** | **Date of switch from organophosphate to neonicotinoid** | **Months of baseline data,**  **count** | **Months of endline data, count** | **Baseline malaria incidence category** | **Mean**  **malaria**  **incidence during organophosphate period, per**  **1,000 person-years** | **Mean malaria incidence during neonicotinoid period, per 1,000 person-years** |
| --- | --- | --- | --- | --- | --- | --- | --- |
| North East | Bunkpurugu -Nakpanduri | Mar-20 | 62 | 34 | Moderate | 222 | 280 |
|  | East Mamprusi | Mar-19 | 50 | 46 | Low | 158 | 317 |
|  | Mamprugu-Moagduri | Mar-19 | 50 | 46 | Low | 189 | 188 |
|  | West Mamprusi | Apr-19 | 51 | 45 | Low | 83.5 | 143 |
|  | Yunyoo-Nasuan | Mar-20 | 62 | 34 | Low | 126 | 257 |
| Northern | Gushiegu***^†^*** | Mar-20 | 38 | 34 | Low | 75.2 | 94.8 |
|  | Karaga***^†^*** | Mar-20 | 38 | 34 | Low | 108 | 155 |
|  | Kumbungu | Mar-20 | 62 | 34 | Low | 154 | 94.6 |
| Upper East | Builsa North* | Apr-19 | 27 | 45 | Moderate | 208 | 256 |
|  | Builsa South* | Apr-19 | 27 | 45 | Moderate | 247 | 388 |
|  | Kasena-Nankana West* | Apr-19 | 27 | 45 | Moderate | 319 | 320 |
| Upper West | Daffiama-Bussie-Issa | Apr-18 | 39 | 57 | Moderate | 306 | 447 |
|  | Jirapa | Apr-18 | 39 | 57 | Moderate | 245 | 277 |
|  | Lambussie | Mar-19 | 50 | 46 | Moderate | 369 | 480 |
|  | Lawra | Mar-19 | 50 | 46 | Moderate | 286 | 405 |
|  | Nadowli-Kaleo | Apr-18 | 39 | 57 | Moderate | 287 | 391 |
|  | Nandom | Apr-19 | 51 | 45 | Moderate | 240 | 332 |
|  | Sissala East | Apr-19 | 51 | 45 | Moderate | 342 | 373 |
|  | Sissala West | Apr-19 | 51 | 45 | Moderate | 364 | 442 |
|  | Wa East | Apr-19 | 50 | 46 | Moderate | 220 | 306 |
|  | Wa Municipal | Apr-19 | 50 | 46 | Moderate | 306 | 209 |
|  | Wa West | Apr-19 | 50 | 46 | Moderate | 273 | 402 |
| ***^†^****IRS was withdrawn in 2012 and re-introduced in 2015 in Kumbungu and 2017 in Gushegu and Karaga.*  **IRS was withdrawn from Upper East region in 2015 and re-introduced in Builsa North, Builsa South, and Kasena-Nankana West districts in 2017.* | | | | | | | |
