# Supplementary material for: Malaria incidence rose following the introduction of neonicotinoid-based IRS in selected districts in northern Ghana: An observational analysis
Source: PLOS Glob Public Health. 2026 Apr 17;6(4):e0005267. doi: 10.1371/journal.pgph.0005267 (PMC13089701; doi:10.1371/journal.pgph.0005267)
Supplement: S4 Table — (DOCX) [file pgph.0005267.s009.docx]

**S4 Table**. **Incidence rate ratios comparing observed malaria incidence to counterfactual malaria incidence modelled using interrupted time series methods**

|  | **Post-change to neonicotinoid-based IRS**  **IRR (95% CI)** |
| --- | --- |
| All districts | 1.26 (1.07, 2.08) |
| Stratified by region |  |
| Northern | 1.76 (1.04, 2.74) |
| Northeast | 1.83 (1.08, 3.40) |
| Upper East | 4.26 (2.17, 8.50) |
| Upper West | 1.25 (0.90, 1.70) |
| Stratified by baseline malaria incidence, grouped by districts |  |
| Low incidence | 1.54 (1.14, 2.23) |
| Moderate incidence | 1.20 (1.02, 1.43) |
