# Supplementary material for: Malaria incidence rose following the introduction of neonicotinoid-based IRS in selected districts in northern Ghana: An observational analysis
Source: PLOS Glob Public Health. 2026 Apr 17;6(4):e0005267. doi: 10.1371/journal.pgph.0005267 (PMC13089701; doi:10.1371/journal.pgph.0005267)
Supplement: S5 Table — (DOCX) [file pgph.0005267.s010.docx]

**S5 Table.**  **Associations between covariates and malaria incidence in the final model pooled across regions.**

| **Variable** | **IRR (95% CI)** |
| --- | --- |
| Maximum temperature, per 10 °C increase***^†^*** | 1.37* (1.07, 1.78) |
| Minimum temperature, per 10 °C increase***^†^*** | 0.83** (0.72, 0.95) |
| Normalized Difference Vegetation Index (NDVI) | 1.02*** (1.01, 1.03) |
| Precipitation, per 100 mm increase***^†^*** | 0.93*** (0.90, 0.97) |
| Post SMC | 1.21* (1.01, 1.45) |
| *<0.05, **<0.01, ***<0.001  ***^†^****IRRs represent the multiplicative change in malaria incidence associated with a 10 °C increase in temperature or a 100 mm increase in precipitation.* | |
