# Supplementary material for: Malaria incidence rose following the introduction of neonicotinoid-based IRS in selected districts in northern Ghana: An observational analysis
Source: PLOS Glob Public Health. 2026 Apr 17;6(4):e0005267. doi: 10.1371/journal.pgph.0005267 (PMC13089701; doi:10.1371/journal.pgph.0005267)
Supplement: Attachment — Submitted filename: Responses to Comments.docx [file pgph.0005267.s012.docx]

**Reviewer #1**: “*This is an interesting analysis that attempts to assess impact of IRS insecticide changes on malaria incidence as reported in passive surveillance data. I agree with the authors that ITS is a great approach to explore this question, however I have some concerns that the complex mix of interventions applied in the study area have been over-simplified and the impact of mass ITN distribution in 2015 and SMC introduction are insufficiently discussed in the paper. This potential bias needs to be unpicked further to justify the authors’ conclusions that increases in incidence are attributable to switch from organophosphate to neonicotinoids.*

*Specifically:*

1. *Authors mention that SMC began in Upper West in 2015, and was then scaled to other regions between 2016 and 2019. It would be useful to include this phased-scale up in one of the figures or tables outlining the intervention mix over time (perhaps a hatching pattern in table S1?) Furthermore, while inclusion of a covariate for SMC is mentioned on line 202, the SMC coefficient value in the fitted model is not reported. I recommend adding a table reporting the coefficient values for the final model, so that the coefficient size and direction for SMC and the environmental variables can be seen by readers*.”

**Response**:

i. A table outlining the intervention mix has been added as a supplementary table “*S2 Table: Timeline for the introduction of different interventions during the study period*”.
ii. The SMC coefficient value in the fitted model has been added to the supplementary file as S5 Table.
2. *“A mass ITN distribution in 2015 is mentioned in the footnote to table S1, but in the manuscript (line 130) you say that ITNs are only given as part of ANC or child welfare visits. This mass distribution could have suppressed transmission in the few years after 2015, potentially over-estimating the impact of organophosphate IRS on malaria incidence. There might be a few ways to try and incorporate the mass ITN distribution in your model: with a covariate that diminishes over time, informed by any local data on ITN durability (insecticide & physical). Another alternative would be to extend your baseline period to include 1-2 years prior to the mass distribution, though I understand this could be difficult as a result of lower data quality prior to 2015. At the least, I feel that this issue should be discussed as a potential limitation”.*

**Response:**

We thank the reviewers for pointing this out. The mass ITN distribution campaign timeline has been corrected in the manuscript and the different channels of distribution clarified (please refer to **Line 136 to 141 pg. 7 of the revised tracked version**)

i. All districts received pyrethroid-only ITN during 2016 mass ITN distribution campaign. From 2018 onwards, however, districts receiving IRS (the study districts) were exempted from subsequent mass ITN campaigns. ITN coverage in the study districts has since 2018 been maintained primarily through continuous distribution channels, including antenatal clinics (for pregnant women) and child welfare clinics (for children under five years of age). please refer to **Line 136 to 141 pg. 7 of the revised tracked version**)
ii. We have included in the manuscript an additional sensitivity analysis with data beginning in 2017 (therefore excluding the two years immediately after the distribution) (S3 Fig). Results were qualitatively similar to the main findings, but less precise (IRR = 1.21, 95% CI 0.90-1.85) **(Line 244 to 247; & line 254 & 257 pg. 12-13 of the revised tracked version**)
iii. We acknowledge that the mass ITN distribution of ITNs may have contributed to reduced vector indoor exposure especially in the first two years post distribution. However, evidence suggests that pyrethroid-only ITNs had limited impact in this setting.
     a. Entomological data from sentinel sites that received ITNs alone between 2016 and 2017 showed no reductions in vector parity, possibly due to high pyrethroid resistance intensity (Dadzie *et al.*, 2016). In districts which did not receive IRS, parity increased significantly from 67% in 2015 to approximately 76% following ITN distribution (Z = −7.36, p < 0.001). In districts where IRS was withdrawn in 2015, vector parity increased sharply from 28% during the IRS implementation to 66% by 2017 (p = 0.001), despite the period coinciding with mass ITN distribution. In contrast, in Kumbungu district, where IRS was reintroduced, parity declined from 55% in 2016 to 40% in 2017, two (Z = 6.77, p < 0.001).
     b. ITN usage was also modest: the 2016 MIS estimated use at 51%, and our 2017 surveys (<https://doi.org/10.1186/s13071-023-05793-2>) found lower ITN usage in IRS communities (37.8%) than in non-IRS (unsprayed) communities (57.3%), despite high ownership.
     c. Importantly, the IRS districts recorded 26% and 58% lower incidence in 2016 and 2017, respectively, compared with neighbouring non-IRS districts. Additionally in Upper East Region, where IRS was withdrawn in 2015 and communities received ITNs; malaria incidence increased by an average of 485% per district (95% CI: 330–640%) compared with 2014 (<https://doi.org/10.1186/s12936-020-03318-1>).

These have been discussed in the main text of paper (**Line 421 to 443**).

3. *“The description of your primary outcome, malaria incidence, requires a bit more detail in lines 179-185. Did you adjust for changes in testing by including testing rate directly in your model? You mention three DHIS2 indicators (suspected, tested suspected and confirmed cases), but it is not clear which were used or how, and I do not think it would be possible to reproduce the analysis based on information provided*”.

**Response:**

We have revised lines 179–185 (now 183 to 195) to clarify the primary outcome and the main indicator used.

As stated in the opening sentence of that paragraph - line 179 *“* ***The primary outcome for this analysis is district-level malaria incidence recorded per 1000 person-years.***  For each district and month, DHIS2 reports three malaria indicators: the number of suspected malaria cases, the number of suspected malaria cases tested, and the number of confirmed malaria cases identified. Malaria incidence was estimated as the number of confirmed malaria cases divided by the population at risk and adjusted for testing.

4. *How did you code Xct for the districts that were not sprayed in 2015 or 2016? Were those district-months dropped from the analysis set, or coded the same as the districts which received organophosphate IRS?*

**Response**:

We have added data on additional sensitivity analyses done to confirm the validity of our conclusions (**Line 244 to 257**).

i. First, we excluded districts that were not sprayed during the 2015–2016 IRS period. The estimated post-intervention effect remained evident, with observed malaria incidence exceeding the counterfactual (IRR = 1.35, 95% CI 1.01–1.78) shown in S2 Fig.
ii. For the second analysis, we restricted the analysis to data from 2017 onwards, when intervention implementation was consistent across districts. The estimated effect was similar in direction but less precise (IRR = 1.21, 95% CI 0.90–1.85) shown in S3 Fig.

Results from these sensitivity analyses have been added as part of the supplementary information (Fig S2-S4) and the data now presented in the main text (**Line 342 to 350**).
Additional suggestions:

5. *Did you consider extending this analysis to a controlled interrupted time series, by including some of the districts in these regions which did not receive any IRS? This could help with disentangling the effect IRS from that of SMC and mass ITN distribution*.

**Response:**
Yes, we considered this approach; however, we were unable to identify suitable contemporaneous control districts. Districts without IRS received other malaria interventions (including PBO nets) around the same period and differed substantially in context (predominantly peri-urban versus rural and differed in ecology), violating key assumptions of controlled ITS (or difference-in-differences) designs.

6. *I would be interested to see a sensitivity analysis that uses just confirmed malaria cases as the outcome, rather than incidence adjusted for testing rates. However, if comment #3 is adequately addressed, this may be redundant*.

**Response:**

As suggested, we conducted a sensitivity analysis using confirmed malaria cases as the outcome. Results were highly consistent with the primary analysis, reflecting the strong correlation between confirmed and testing-adjusted cases. These findings are now included in the Supplementary Information (S4 Fig) and referenced in the Results (**Line 342 to 350*)***.

7. *Do you have any indication of if treatment seeking for fever changed over the study period, or indeed treatment seeking more broadly? In particular, this study period includes the COVID-19 pandemic, when treatment seeking could have altered from the usual levels. All-cause outpatient attendance data in DHIS2 could help to assess this*.

**Response:**
Yes. Analyses of routine surveillance data show that treatment seeking and health-facility attendance declined during the COVID-19 pandemic. All outpatient department (OPD) visits declined by up to 27% during the second and third quarters of 2020 compared with the 2015–2019 average, coinciding with movement restrictions and reduced access to health services (Heuschen *et al.*, 2022).

Declines were also reported in suspected malaria cases (14.1%) and malaria admissions (21.6%) at the national level during the same period (Malm *et al.*, 2024).

Consequently, any pandemic-related bias introduced would tend to underestimate malaria burden, making our findings conservative. The magnitude of post-NN IRS incidence may have been greater than observed.

We have expanded the discussion to address the implications of these changes in health-seeking behaviour during the COVID-19 pandemic (**Line 401 to 407)**.

8. In table S2, what does * indicate?

**Response:**
This has been corrected.
