# Supplementary material for: Malaria incidence rose following the introduction of neonicotinoid-based IRS in selected districts in northern Ghana: An observational analysis
Source: PLOS Glob Public Health. 2026 Apr 17;6(4):e0005267. doi: 10.1371/journal.pgph.0005267 (PMC13089701; doi:10.1371/journal.pgph.0005267)
Supplement: Attachment — Submitted filename: Responses_to_Comments_auresp_2.docx [file pgph.0005267.s013.docx]

Reviewer #1*: Thank you for the clear and detailed responses to my previous comments. I’m satisfied with the changes made and wish to congratulate the authors on a strong manuscript.

In reading through the revised manuscript, I have just two minor edits to flag:*

- *I suggest checking content in table S2. The colour and labels appear to be in a different order for the Upper West Region than for the other regions, which I assume is a copy/paste error in the left-most column.*

**Response**:

Thank you for flagging this typo. The table has been corrected. The colour and labels for upper West Region in ‘*S2 Table’* has been corrected. The table is now *S1 Table* due to align with the citations in the main manuscript.

- *Recommend adding units for precipitation (mm?) and temperature to table S5 so that the effect sizes can be easily interpreted. You may also want to consider rescaling or standardizing precipitation so that the IRR is more interpretable.*

**Response**:

Thank you for the suggestion.

We have now rescaled the climatic covariates to improve interpretability of the incidence rate ratios. Temperature was modelled per 10 °C increase and precipitation per 100 mm increase. This has been added to the text and the corresponding units added to S5 Table to facilitate interpretation of effect sizes.
